# Online Patient Education Materials for Pediatric Septic Arthritis Exceed Recommended Reading Levels: A Readability Analysis

**DOI:** 10.7759/cureus.101617

**Published:** 2026-01-15

**Authors:** Nicholas J Pettinelli, Madison C Blackwell, Mark Schwartz

**Affiliations:** 1 Trauma Institute, Saint Francis Health System, Tulsa, USA; 2 Orthopaedic Surgery Department, Oklahoma State University Center for Health Sciences, Tulsa, USA; 3 Pediatric Orthopaedics Department, Children's Hospital at Saint Francis, Tulsa, USA

**Keywords:** health literacy, orthopedic surgery, patient education materials, pediatric septic arthritis, readability

## Abstract

Background: Pediatric septic arthritis is a time-sensitive orthopedic emergency requiring prompt recognition and treatment to avoid serious morbidity. Families often seek information online, yet prior studies show patient education materials (PEMs) in orthopedics frequently exceed recommended readability standards (≤6th-grade level).

Purpose: This study aims to evaluate the readability of online PEMs on pediatric septic arthritis from top-ranked US pediatric orthopedic hospitals and assess alignment with readability guidelines.

Methods: In July 2025, websites of the top 25 US pediatric orthopedic hospitals were searched for PEMs on “septic arthritis” in children. Hospitals were included if they hosted a dedicated PEM ≥100 words. Text was extracted, cleaned of non-narrative elements, and analyzed with eight readability metrics: Flesch-Kincaid Grade Level (FKGL), Gunning Fog Index, Simple Measure of Gobbledygook (SMOG), Coleman-Liau Index, Automated Readability Index (ARI), Flesch Reading Ease Score (FRES), Ford, Caylor, Sticht (FORCAST) formula, and Dale-Chall Readability Score. Descriptive statistics were summarized, and FKGL was correlated with hospital rank using Spearman’s test.

Results: Of 25 hospitals, 12 (48%) hosted qualifying PEMs. The mean readability was grade 10.6, above the recommended sixth-grade level; none achieved ≤6. Seven institutional PEMs were written at an average reading level significantly above the eighth-grade reading level (p < 0.01). Five PEMs (41.7%) were written at or below the eighth-grade level, largely from identical third-party content. Mean FRES was 51, reflecting “somewhat challenging” readability. Higher-ranked hospitals trended toward worse FKGL scores, but correlation was nonsignificant (ρ = -0.32; p = 0.31).

Conclusions: Nearly half of the top hospitals lacked PEMs on pediatric septic arthritis, and available content largely exceeded recommended readability. Adoption of plain-language guidelines, external audits, or artificial intelligence (AI)-assisted editing may enhance accessibility and equity.

## Introduction

Pediatric septic arthritis is an acute bacterial infection of the joint that constitutes a true orthopedic emergency. Although relatively uncommon in high-income settings, with an incidence of approximately 4-10 per 100,000 children annually, it remains a critical condition due to the risk of rapid joint destruction, growth disturbances, and long-term disability if not promptly diagnosed and treated [1,2]. Management of pediatric septic arthritis entails urgent drainage of the affected joint, combined with empiric intravenous antibiotic therapy tailored to culture results [3,4]. When treated promptly, outcomes are excellent; however, delays can result in significant morbidity, including growth disturbances, and chronic disability [4,5].

Patients and their families are more dependent on online medical educational resources than ever before [6,7]. While the ubiquity of mobile devices and computers makes accessing health information easy, the ability of patients to comprehend online resources is dependent upon reader literacy. Prior research has suggested that online material be written at a sixth-grade reading level in an effort to improve health literacy, enhance patient understanding, and ultimately better patient outcomes [8,9]. Further, census information has informed that the average US adult reads at an eighth-grade level [10].

As a result, there has been extensive research targeted at readability analysis used in numerous orthopedic subspecialties to evaluate the quality and comprehensibility of patient education materials (PEMs) [6,7,11-13]. Readability is the process of evaluating the difficulty of a written text for a given audience and measures the complexity of language while assigning an education level required to understand a given text. Depending upon the formula used, various reading scores place emphasis on different metrics and include Flesch-Kincaid Grade Level (FKGL), Gunning Fog Index, Simple Measure of Gobbledygook (SMOG) Index, Coleman-Liau Index, Automated Readability Index (ARI), Flesch Reading Ease Score (FRES), Ford, Caylor, Sticht (FORCAST) formula, and Dale-Chall Readability Score.

To our knowledge, no previous study has explored the readability of PEMs pertaining to pediatric septic arthritis. The purpose of this study was to investigate the readability of PEM available to patients online at leading orthopedic institutions in the United States. It was hypothesized that online educational material for pediatric septic arthritis available on the webpages of top academic centers is above the recommended reading level of the majority of adults in the United States.

## Materials and methods

Identification of institutions

In July of 2025, we systematically searched for online educational materials related to pediatric septic arthritis hosted on the websites of leading pediatric orthopedic institutions. Twenty-five institutions were identified based on the 2024-2025 US News and World Report ranking for pediatric orthopedic surgery [14]. Using the Google search engine, we queried each institution by entering the hospital name followed by the keywords “septic arthritis” and “children” (e.g., “XYZ Children’s Hospital septic arthritis children”). Searches were performed in a private browser window to minimize personalization of results.

For each hospital, web pages were included if they had a dedicated PEM page on pediatric septic arthritis. If no relevant page was identified, the hospital was recorded as having no available information. Based upon prior readability studies and the limited reliability of readability scores on short segments of text, web pages with fewer than 100 words of content specific to pediatric septic arthritis were excluded from further readability analysis. An agreement was required between two independent reviewers for the final inclusion of web pages for readability analysis.

Readability assessment

Patient education materials (PEM) relevant to pediatric septic arthritis from each website were catalogued and converted into a text-only format via Microsoft Word (Microsoft Corp., Redmond, WA) to exclude any figures, videos, tables, disclaimers, acknowledgements, citations, references, and hyperlinks. Reformatted patient education files were then analyzed for readability utilizing Readability Studio Professional Edition software version 2019 (Oleander Software) (http://www.oleandersolutions.com/).

Readability was assessed using eight standard indices via their associated formulas shown in Table 1.

**Table 1:** Readability assessments and formulas FKGL: Flesch-Kincaid Grade Level, FRES: Flesch Reading Ease Score, SMOG: Simple Measure of Gobbledygook, ARI: Automated Readability, FORCAST: Index, Ford, Caylor, Sticht

Readability Assessment	Formula
FKGL	$0.39 \times (\frac{totalwords}{total sentences}) + 11.8 \times (\frac{total syllables}{total words}) - 15.59$
FRES	$206.835 - (1.015 \times \text{mean # of words per sentence}) - (84.6 \times \text{mean # of syllables per word})$
Gunning Fog Index	$0.4 \times \left( \frac{\text{mean # words}}{\text{mean # of sentences}} + 100 \times \frac{\text{mean # words with }\ge 3\text{ syllables}}{\text{100 words}} \right)$
Coleman-Liau Index	$0.0588 \times \left( \frac{\text{mean # of letters}}{\mathrm{word}} \right) - 0.296 \times \left( \frac{\text{mean # of sentences}}{100\ \mathrm{words}} \right)$
SMOG Index	$1.043 \times \sqrt{ (\text{# of words with }\ge 3\text{ syllables}) \times \left(\frac{30}{\text{# of sentences}}\right) + 3.1291 }$
ARI	$4.71 \times \left( \frac{\mathrm{letters}}{\mathrm{words}} \right) + 0.5 \times \left( \frac{\mathrm{words}}{\mathrm{sentences}} \right) - 21.43$
FORCAST	$20 - \left( \frac{\text{# of single syllable words in 150 word sample}}{10} \right)$
New Dale-Chall Index	$0.496 \times \left( \frac{\text{mean # of words}}{\text{mean # of sentences}} \right) + 0.1579 \times \left( \frac{\text{unfamiliar words}}{\text{mean # of words}} \right) + 3.6365$

All algorithms ascertained the reading grade level (RGL) of the text file, with the exception of the Flesch Reading Ease Score (FRES). RGLs demonstrate the level of US-based education necessary to read and understand a selection of texts.

For each selection of text, the seven RGL tests generated seven RGL scores, as well as a mean RGL. In contrast, the FRES formula expresses readability as an index score that is based on sentence length and the number of syllables. The score ranges from 0 to 100, with higher scores indicating an easier reading level. The readability categories for the Flesch-Kincaid reading ease scores are defined as follows: scores from 90 to 100 signify texts that are very easy to understand, 80 to 89 denote easy reading, 70 to 79 are considered fairly easy, 60 to 69 represent standard difficulty, 50 to 59 are somewhat challenging, 30 to 49 categorize texts as difficult, and scores between 0 and 29 are labeled as confusing.

Data analysis

Data were entered and analyzed using Microsoft Excel 2010 and Jamovi cloud-based open access statistics (The jamovi project (2025). jamovi (Version 2.6) [Computer Software]. Retrieved from https://www.jamovi.org) [15]. Descriptive statistics were used to summarize the availability of resources on institution websites and readability scores. Readability scores were reported as means ± standard deviations (SD). The seven reading grade level scores were averaged for each of the 12 institutions with PEM. The means from each institution were compared to a reference mean eighth-grade reading level with a two-tailed, one-sample t*-*test. We also tested the relationship between hospital rank and Flesch-Kincaid readability grade levels using Spearman’s rank correlation.

## Results

In total, 12 of the top 25 (48%) pediatric orthopedic hospitals in the country (as designated by the 2025 US News and World Report Best Children’s Hospitals for Orthopedics Ranking) [14] were identified to have patient-/caregiver-directed PEM relevant to pediatric septic arthritis. Thirteen of the other top 25 (52%) pediatric orthopedic hospitals in the United States had no information available for patients or their families on pediatric septic arthritis.

**Figure 1:**
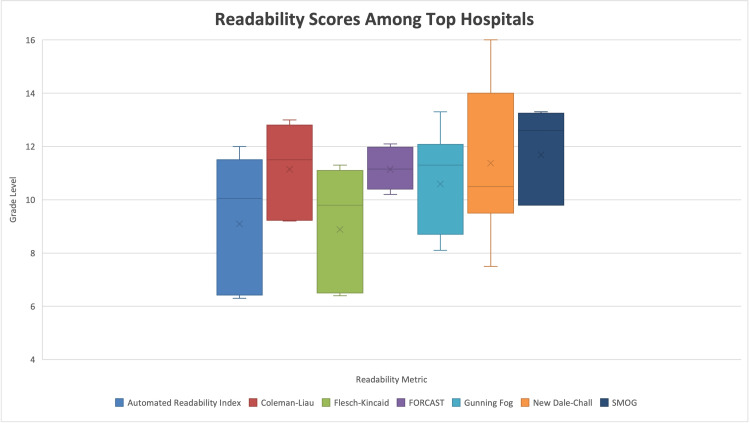
Distribution of institution webpage readability by analysis tool Note: Sixth-grade reading level is what is suggested by the CDC. Eighth-grade reading level corresponds to the reading level of the average US adult. FORCAST: Ford, Caylor, Sticht, SMOG: Simplified Measure of Gobbledygook

**Figure 2:**
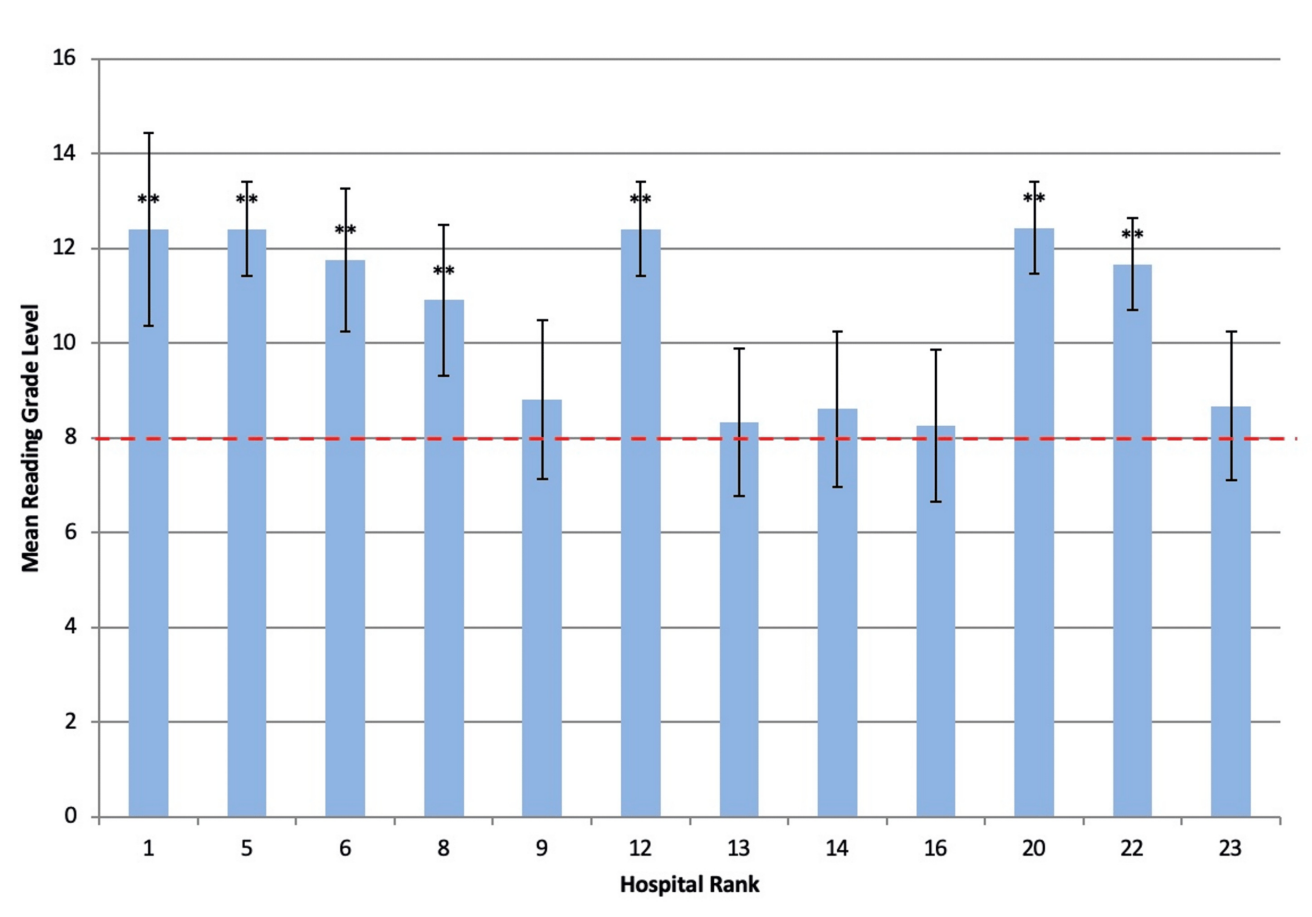
Average reading grade level for each ranked institution with available PEM Error bars denote standard deviation. “**” represents a significant difference (p < 0.05) from the suggested eighth-grade reading level suggested by the NIH (red dotted line). PEM: patient education material

**Figure 3:**
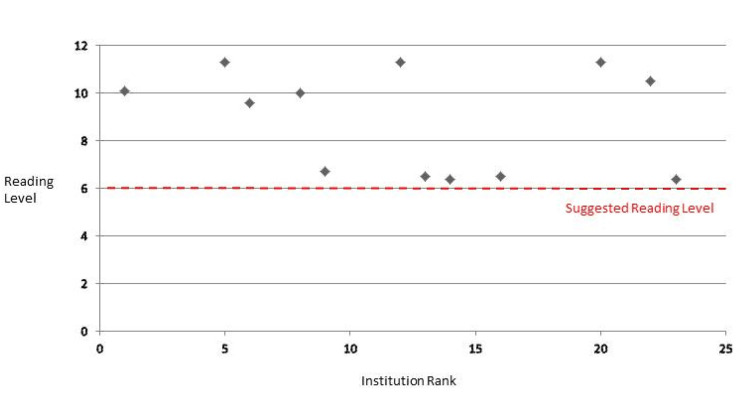
Flesch-Kincaid reading grade level for online PEM at leading pediatric orthopedic institutions relative to the suggested audience reading level PEM: patient education material

Readability scores were calculated for all web pages that met the inclusion criteria. Readability analysis scores and their aggregate averages are shown in Table 2. The overall readability level across hospitals was 10.6, indicating a high school sophomore grade level required to fully comprehend written information. The lowest averaged grade level required to understand online materials was an average score of 8.3 ± 1.5. The highest average RGL was 12.4 ± 1.9 (Table 2). None of the institution’s web pages had reading levels that corresponded to below sixth grade reading level, as suggested by the CDC (Figure 1) [8]. When compared to a value of 8.0, the mean reading grade level of 7 of the 12 institutions were found to be significantly above the average eighth-grade reading level of US adults (p < 0.05) (Figure 2, Table 2). The other five institutions were found to be slightly above an eighth-grade reading level; however, this was not significant (p > 0.05) (Figure 2, Table 2). These five institutions (ranked 9, 13, 14, 16, and 23) had RGL scores comparable to a sixth-grade reading level when appraised by the Automated Readability Index and the Flesch-Kincaid score (Figure 3). The average Flesch Reading Ease score (FRES) across all 12 institutions’ included websites was 51, indicating a somewhat challenging text (Table 3). The highest FRES was 67, indicating standard difficulty for the average US adult. No institution achieved an FRES indicating fairly easy-to-read text.

**Table 2:** Readability index scores of pediatric septic arthritis PEM among the top 25 ranked pediatric orthopedic surgery hospitals The average reading grade levels for each institution were compared to a reference mean eighth-grade reading level with a two-tailed, one-sample t-test. T-statistic and p-values are shown. p-values < 0.05 were deemed significant. PEM: patient education material, ARI: Automated Readability Index, SMOG: Simplified Measure of Gobbledygook, FORCAST: Ford Caylor Sticht, SD: standard deviation

Hospital rank	ARI	Coleman-Liau	Flesch-Kincaid	FORCAST	Gunning Fog	New Dale-Chall	SMOG	Average ± SD	t-statistic	p-value
1	10.2	12.9	10.1	11.6	13	16	13	12.4 ± 1.9	5.7	<0.001
5	12	12.8	11.3	12	11.5	14	13.3	12.4 ± 0.9	11.8	<0.001
6	10.5	13	9.6	11.9	11.1	14	12.2	11.8 ± 1.4	6.6	<0.001
8	9.9	10.4	10	10.2	13.3	9.5	13.1	10.9 ± 1.5	4.9	<0.01
9	6.4	9.5	6.7	11	8.8	9.5	9.8	8.8 ± 1.6	1.3	0.248
12	12	12.8	11.3	12	11.5	14	13.3	12.4 ± 0.9	11.8	<0.001
13	6.5	9.2	6.5	10.4	8.5	7.5	9.8	8.3 ± 1.4	0.6	0.582
14	6.3	9.2	6.4	10.4	8.7	9.5	9.8	8.6 ± 1.5	1.0	0.358
16	6.3	9.2	6.5	10.4	8.1	7.5	9.8	8.3 ± 1.5	0.4	0.686
20	11.8	12.8	11.3	12.1	11.7	14	13.3	12.4 ± 0.9	12.1	<0.001
22	10.6	12.6	10.5	11.3	12.2	11.5	13	11.7 ± 0.9	10.1	<0.001
23	6.6	9.3	6.4	10.4	8.7	9.5	9.8	8.7 ± 1.5	1.1	0.301

**Table 3:** Hospital rank and Flesch Reading Ease Score The score ranges from 0 to 100, with higher scores indicating an easier reading level. Scores from 90 to 100 signify texts that are very easy to understand, 80 to 89 denote easy reading, 70 to 79 are considered fairly easy, 60 to 69 represent standard difficulty, 50 to 59 are somewhat challenging, 30 to 49 categorize texts as difficult, and scores between 0 and 29 are labeled as confusing.

Hospital rank	Flesch Reading Ease Score
1	46
5	45
6	48
8	54
9	62
12	45
13	66
14	67
16	66
20	44
22	46
23	67

We evaluated the relationship between hospital academic rank and Flesch-Kincaid grade-level readability scores (Table 4) for the 12 top institutions using Spearman’s rank correlation. The Spearman correlation coefficient was -0.32 (p = 0.31), indicating a weak, negative, and statistically nonsignificant association between hospital rank and Flesch-Kincaid score. The median Flesch-Kincaid grade level was approximately 9.8, which is above the recommended sixth-grade level and also above the average US adult reading level of eighth grade.

**Table 4:** Average Flesch-Kincaid score of each of the 12 institutional PEM analyzed by hospital rank PEM: patient education material

Hospital rank	Flesch-Kincaid score
1	10.1
5	11.3
6	9.6
8	10.0
9	6.7
12	11.3
13	6.5
14	6.4
16	6.5
20	11.3
22	10.5
23	6.4

## Discussion

The major finding of this study is that of the 48% top pediatric orthopedic institutions that have caregiver-directed information regarding pediatric septic arthritis, the aggregate reading-grade level of these materials consistently exceeds the sixth-grade standard suggested by the CDC [9]. Of the institutions, 58% had PEM at an RGL significantly higher than the eighth grade, aligning more with an 11th-grade reading level. The other 42% of the institutions analyzed presented content at or above the eighth-grade average reading level of US adults, although this finding was nonsignificant [10]. However, Flesch-Kincaid scores from institutions 9, 13, 14, 16, and 23 demonstrated a sixth-grade reading level. Higher-ranked hospitals tended to have slightly higher Flesch-Kincaid scores (worse readability), but this trend did not reach statistical significance (ρ = -0.32; p = 0.31).

The readability analysis tools utilized in this study have previously demonstrated a high degree of collinearity in the literature [16]. In the past, two indices that have demonstrated the highest degree of collinearity were the SMOG Index and FK scores. Despite this, the SMOG Index from these same institutions averaged a ninth-grade reading level. Our team attributes this discrepancy to a difference in how these scores are calculated. SMOG assesses text for large words with three or more syllables irrespective of sentence length, while FK uses a mix of sentence and word length to judge the difficulty of text. We suspect that in the text analyzed from these select institutions, sentence length was appropriate to an RGL of 6, but the words used were not.

Orthopedic-related PEM being too complex for the general population is not a new problem. Early studies have found that information disseminated via the American Academy of Orthopaedic Surgeons (AAOS) and Pediatric Orthopaedic Society of North America (POSNA) webpages averaged 9th-11th grade reading levels as early as 2008 [6]. This pattern has been echoed by several recent systematic reviews of PEMs [17,18] specific to pediatric orthopedics. A recent evaluation of available information regarding adult septic arthritis found variable quality from online sources, with PEM from academic centers being too difficult to read for the general public [19]. Our results, which span multiple institutions and readability tools, confirm that there is still misalignment between institutional online health materials and the literacy needs of patients and caregivers. However, the ~42% of institutions presenting content at or slightly above an eighth-grade average reading level may suggest that some progress has been made since earlier alarms about readability were raised.

The public health implications of patient-facing online materials are substantial: roughly 36% of US adults have “basic” or “below basic” health literacy [10], and limited literacy is associated with poorer health outcomes [8]. For families navigating pediatric septic arthritis, an urgent, technically complex diagnosis requiring prompt recognition and treatment [4], the cognitive burden imposed by difficult-to-read text may widen disparities in care.

Better readability does not guarantee comprehension, but it is a necessary first step. Encouragingly, emerging technologies show the potential to offer partial solutions. Artificial intelligence (AI) large language models have been shown to lower the readability of pediatric orthopedic PEMs by one to two grade levels without altering meaning [20]. Hospitals should consider integrating such tools, alongside plain-language checklists (e.g., CDC’s Simply Put guide [9]) and iterative user testing. In the current study, the text extracted from the webpages of ranked institutions 9, 13, 14, 16, and 23 had nearly identical content prepared by the same third-party resource. These institutions’ average PEM performed close to the eighth-grade reading level, the lowest RGL for institutions analyzed, suggesting that institutions may increase the readability of their PEM through third-party audits. This methodology, used in conjunction with the aforementioned tools, could be sufficient to aid in bridging the knowledge gap between academic institutions and their intended audience.

Strengths and limitations

The strengths of the current study include the use of seven readability indices and institution-level reporting, providing a granular view of variability across available PEM from top academic centers. Limitations mirror those of earlier readability work: formulas approximate grade level from syllable and sentence counts and do not capture domain knowledge or cultural nuance; we only analyzed English text, potentially under-representing resources available in other languages; webpage content is dynamic, so our snapshot may not reflect future updates; and we did not assess information quality, accuracy, or usability.

Future directions

Prospective studies should examine whether readability improvements translate into measurable gains in caretakers’ knowledge, time to presentation, and treatment adherence for pediatric septic arthritis. Parallel evaluation of the incorporation of visual aids, accessibility features, and non-English materials is also warranted. Finally, incorporating patient and caregiver feedback during content development could ensure that materials meet real-world literacy needs.

## Conclusions

Despite long-standing gaps in health literacy that may potentiate poorer health outcomes, nearly half of the 25 top-ranked pediatric orthopedic institutions lacked available patient-facing information on septic arthritis. Of the pediatric orthopedic institutions that did provide PEM, a majority continue to present information pertaining to pediatric septic arthritis above recommended reading levels and significantly above the average reading levels of US adults. We endorse several solutions to bridging the gap in readability: leveraging modern tools such as large language models with the assistance of generative AI technologies to align web content with recommended standards; utilizing plain-language principles and the use of guides; and utilizing third-party, non-medical entities to limit medical jargon and simplify information without diluting the take-home message of PEM. Improving readability remains an achievable, evidence-supported intervention to enhance patient understanding and patient-centered care.

## Appendices

The descriptive statistics for each institution’s PEM are shown in Table 5.

**Table 5:** Descriptive statistics for each institution’s PEM analyzed PEM: patient education material, ARI: Automated Readability Index, FORCAST: Ford Caylor Sticht, SMOG: Simplified Measure of Gobbledygook, SD: standard deviation

Hospital rank	ARI	Coleman-Liau	Flesch-Kincaid	FORCAST	Gunning Fog	New Dale-Chall	SMOG	Average ± SD	Average	SD	Median	Min	Max	25th percentile	75th percentile
1	10.2	12.9	10.1	11.6	13	16	13	12.4 ± 1.9	12.4	1.9	12.9	10.1	16	10.9	13.0
5	12	12.8	11.3	12	11.5	14	13.3	12.4 ± 0.9	12.4	0.9	12	11.3	14	11.8	13.1
6	10.5	13	9.6	11.9	11.1	14	12.2	11.8 ± 1.4	11.8	1.4	11.9	9.6	14	10.8	12.6
8	9.9	10.4	10	10.2	13.3	9.5	13.1	10.9 ± 1.5	10.9	1.5	10.2	9.5	13.3	10.0	11.8
9	6.4	9.5	6.7	11	8.8	9.5	9.8	8.8 ± 1.6	8.8	1.6	9.5	6.4	11	7.8	9.7
12	12	12.8	11.3	12	11.5	14	13.3	12.4 ± 0.9	12.4	0.9	12	11.3	14	11.8	13.1
13	6.5	9.2	6.5	10.4	8.5	7.5	9.8	8.3 ± 1.4	8.3	1.4	8.5	6.5	10.4	7.0	9.5
14	6.3	9.2	6.4	10.4	8.7	9.5	9.8	8.6 ± 1.5	8.6	1.5	9.2	6.3	10.4	7.6	9.7
16	6.3	9.2	6.5	10.4	8.1	7.5	9.8	8.3 ± 1.5	8.3	1.5	8.1	6.3	10.4	7.0	9.5
20	11.8	12.8	11.3	12.1	11.7	14	13.3	12.4 ± 0.9	12.4	0.9	12.1	11.3	14	11.8	13.1
22	10.6	12.6	10.5	11.3	12.2	11.5	13	11.7 ± 0.9	11.7	0.9	11.5	10.5	13	11.0	12.4
23	6.6	9.3	6.4	10.4	8.7	9.5	9.8	8.7 ± 1.5	8.7	1.5	9.3	6.4	10.4	7.6	9.7

## References

[1] Brown DW, Sheffer BW (2019). Pediatric septic arthritis: an update. Orthop Clin North Am.

[2] Montgomery NI, Epps HR (2017). Pediatric septic arthritis. Orthop Clin North Am.

[3] Erkilinc M, Gilmore A, Weber M, Mistovich RJ (2021). Current concepts in pediatric septic arthritis. J Am Acad Orthop Surg.

[4] Mallet C, Ilharreborde B, Caseris M, Simon AL (2025). Treatment of septic arthritis of the hip in children. Orthop Traumatol Surg Res.

[5] Nannini A, Giorgino R, Bianco Prevot L (2023). Septic arthritis in the pediatric hip joint: a systematic review of diagnosis, management, and outcomes. Front Pediatr.

[6] Badarudeen S, Sabharwal S (2008). Readability of patient education materials from the American Academy of Orthopaedic Surgeons and Pediatric Orthopaedic Society of North America web sites. J Bone Joint Surg Am.

[7] Stelzer JW, Wellington IJ, Trudeau MT, Mancini MR, LeVasseur MR, Messina JC, Mazzocca AD (2022). Readability assessment of patient educational materials for shoulder arthroplasty from top academic orthopedic institutions. JSES Int.

[8] (2025). The extent and associations of limited health literacy. Health Literacy: A Prescription to End Confusion.

[9] (2025). Simply Put: A guide for creating easy-to-understand materials. https://stacks.cdc.gov/view/cdc/11938.

[10] Kutner M, Greenberg E, Jin Y, Paulsen C (2006). The Health Literacy of America's Adults: Results from the 2003 National Assessment of Adult Literacy. NCES 2006-483. Natl Cent Educ Stat. Published online September.

[11] Schumaier AP, Kakazu R, Minoughan CE, Grawe BM (2018). Readability assessment of American Shoulder and Elbow Surgeons patient brochures with suggestions for improvement. JSES Open Access.

[12] Polishchuk DL, Hashem J, Sabharwal S (2012). Readability of online patient education materials on adult reconstruction web sites. J Arthroplasty.

[13] Bluman EM, Foley RP, Chiodo CP (2009). Readability of the patient education section of the AOFAS website. Foot Ankle Int.

[14] (2025). Best children's hospitals for orthopedics. https://health.usnews.com/best-hospitals/pediatric-rankings/orthopedics.

[15] (2025). jamovi: open statistical software for the desktop and cloud. https://www.jamovi.org/.

[16] Michel C, Dijanic C, Abdelmalek G, Sudah S, Kerrigan D, Gorgy G, Yalamanchili P (2023). Readability assessment of patient educational materials for pediatric spinal conditions from top academic orthopedic institutions. J Child Orthop.

[17] Hecht CJ 2nd, Burkhart RJ, McNassor R, Mistovich RJ (2023). Readability of online patient educational materials in pediatric orthopaedics: a systematic review. J Pediatr Orthop.

[18] Ó Doinn T, Broderick JM, Abdelhalim MM, Quinlan JF (2021). Readability of patient educational materials in pediatric orthopaedics. J Bone Joint Surg Am.

[19] Golgelioglu F, Canbaz SB (2023). From quality to clarity: evaluating the effectiveness of online ınformation related to septic arthritis. J Orthop Surg Res.

[20] Nian PP, Williams CJ, Senthilnathan IS (2025). Currently available large language models are moderately effective in improving readability of English and Spanish patient education materials in pediatric orthopaedics. J Am Acad Orthop Surg.

